# Cytomegalovirus Infection of the Gastrointestinal Tract in Patients on Hemodialysis: A Case Report and Literature Review

**DOI:** 10.7759/cureus.64129

**Published:** 2024-07-09

**Authors:** Toshiyuki Nakanishi, Kazuhiro Ishikawa, Yusuke Ohashi, Takuya Fujimaru, Mori Nobuyoshi

**Affiliations:** 1 Department of Internal Medicine, Nerima Hikarigaoka Hospital, Tokyo, JPN; 2 Department of Infectious Diseases, St. Luke's International Hospital, Tokyo, JPN; 3 Department of Nephrology, St. Luke's International Hospital, Tokyo, JPN

**Keywords:** gastroenteritis, endoscopy, biopsy, immunocompetence, renal dialysis, cytomegalovirus (cmv)

## Abstract

Cytomegalovirus (CMV) infection is widespread in immunocompromised people, and several cases of CMV infections of the gastrointestinal (GI) tract have been reported in these individuals. We present a case of an immunocompetent patient on hemodialysis (HD) who developed CMV colitis. We also conducted a review of the literature on CMV GI tract infections among patients with chronic kidney disease undergoing dialysis. A 46-year-old man with a history of end-stage renal disease and undergoing HD developed severe diarrhea and hematochezia. A colonoscopy revealed ulcers, and CMV infection was identified in the biopsy sample. We successfully treated the patient with valganciclovir for 2 months. Our review of the literature yielded 21 articles and 24 cases of CMV GI tract infection in patients undergoing dialysis, including the current case. Hematochezia and diarrhea were purported to serve as indicators of CMV GI tract infection among patients on dialysis. Thus, clinicians should suspect CMV infection of the GI tract in dialysis patients, who experience unexplained bloody diarrhea, and promptly perform a GI endoscopy and biopsy.

## Introduction

Cytomegalovirus (CMV) infection is common in immunosuppressed individuals. However, CMV reactivation can sometimes occur without immunosuppression, with blood transfusion being a risk factor for developing CMV infection among immunocompetent patients [1,2]. Although CMV can put several organ systems at risk, the gastrointestinal (GI) tract is affected most frequently in immunocompetent patients [3]. A retrospective cohort study reported that chronic kidney disease (CKD) increases the risk of CMV enteritis [4], while another revealed that even in CKD patients without the presence of overt immunodeficiency, the GI tract is the most common site of involvement [5]. Furthermore, a retrospective cohort study found that the majority of immunocompetent individuals with GI CMV disease had CKD of stage III or more and that this was significantly more common in immunocompetent rather than immunocompromised patients (62.9 vs. 24.2%, P<0.001) [6], and a literature review of 44 immunocompetent patients with CMV colitis found eight of these patients had renal failure, which was considered one of the comorbidities affecting immune function [7].

Thus, a relationship likely exists between CMV reactivation in the GI tract and CKD, particularly in the dialytic state. Here, we present a case of CMV colitis in an immunocompetent patient undergoing hemodialysis (HD) with end-stage renal disease (ESRD). We also performed a literature review and identified the characteristics of GI tract infections with CMV among patients with ESRD receiving HD or peritoneal dialysis.

## Case presentation

**Figure 1:**
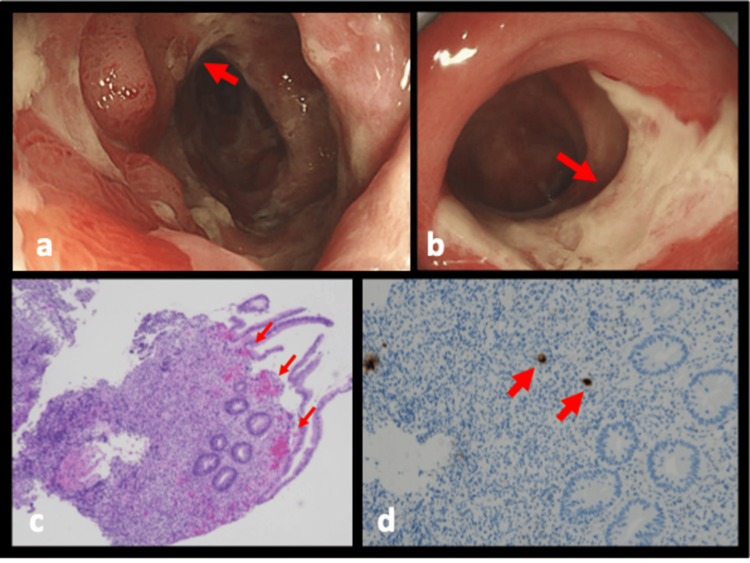
Colonoscopy images Initial colonoscopy (upon admission) (a) reveals erosions and small well-defined ulcers (arrow), consistent with CMV colitis. The follow-up colonoscopy (b) identified scarring and a healed ulcer (arrow). Hematoxylin-eosin staining (×4, c) revealed inflammation of the colon (arrow) , and immunohistochemical staining (×10, d) detected CMV-positive intranuclear inclusions (arrow). CMV: cytomegalovirus

A 46-year-old man with a 9-year history of ESRD, being treated with HD was transferred to our emergency department with complaints of diarrhea and hematochezia. One week before the transfer, he was hospitalized for 4 days with watery diarrhea. During admission, the patient was receiving levofloxacin (500 mg every 48 h) and ceftriaxone (1 g every 24 h) for the possible diagnosis of bacterial gastroenteritis. He stated he had not eaten raw poultry or eggs or traveled abroad recently, and had no history of HIV infection or blood transfusion. His ESRD was due to diabetic kidney disease. The patient was hypotensive upon admission, and a physical examination revealed tenderness in the lower middle abdomen. Laboratory tests showed elevated white blood cell (WBC) count, low hemoglobin, and elevated levels of C-reactive protein (CRP) and creatinine (Table 1). Although differential diagnosis included Campylobacter, Salmonella, and Clostridioides difficile (CD) infection, stool culture did not reveal any pathogenic bacteria, and CD toxin A/B or glutamate dehydrogenase was negative. A colonoscopy revealed erosions and small ulcers around the hepatic flexure (Figure 1a). A tissue biopsy was performed, and immunohistochemical analysis detected CMV-positive cells (Figures 1c-1d). On this basis, the patient was diagnosed with CMV colitis. There were no obvious findings on physical examination suggesting the other CMV direct effects, such as retinitis, ventriculitis, and pneumonia. We administered oral valganciclovir (VGCV, 225 mg) three times a week, and the watery diarrhea decreased. A follow-up colonoscopy was performed a month after treatment initiation. There were still multiple ulcers in the colon (Figure 1b), although the repeat biopsy was CMV-negative. Blood investigation showed normalized WBC count and CRP. He was treated with oral VGCV for 8 weeks. There were no signs of relapse at the 3-month follow-up.

**Table 1:** Results of blood test on admission ALT: alanine transaminase; AST: aspartate transferase; GGTP: gamma-glutamyl transpeptidase

Test	Reference range	Results
White blood cells	4.0-10 x 10^3^/μL	12.8
Neutrophils	40-70%	85
Lymphocytes	20-45%	9
Monocytes	5.0-12%	6
Hemoglobin	12.0-17.0 x g/dL	13.3
Hematocrit	40.0-51.0%	43.2
Platelets	150-400 x 10^3^/μL	216
Sodium	136-146 mmol/L	134
Potassium	3.50-5.10 mmol/L	4.7
Chloride	98-106 mmol/L	99
Urea	2.5-6.6 mmol/L	29.9
Creatinine	72-127 μmol/L	744
ALT	<40 U/L	8
AST	<42 U/L	9
Bilirubin	<17 μmol/L	0.5
GGTP	7-50 U/L	33
Albumin	35-52 g/L	29
C-reactive protein	<5 mg/L	247

## Discussion

**Figure 2:**
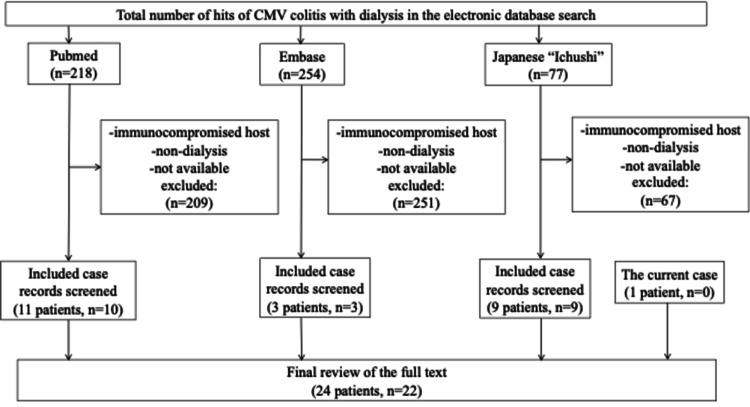
Flowchart of the literature review process. Three authors (T.N., I.K., and Y.O.) independently reviewed the relevant titles and abstracts in the database records, retrieved full texts for eligibility assessment, and extracted information from these cases. We performed a search using the keywords “cytomegalovirus,” “CMV,” “esophagitis,” “gastritis,” “colitis,” “enterocolitis,” “gastroenteritis,” “renal dialysis,” “hemodialysis,” “hemodialysis,” “dialysis,” “chronic,” “kidney,” “renal,” and “nephron” in the electronic databases PubMed, Embase, and Ichushi from inception until May 31, 2022. We excluded immunocompromised patients with HIV infection, recipients of solid organ or hematopoietic stem cell transplantation, those receiving immunosuppression therapy, and those who had not been treated with dialysis before CMV infection. A total of 22 articles and 24 cases of GI CMV infection among dialysis patients were included. CMV: cytomegalovirus; HIV: human immunodeficiency virus; GI: gastrointestinal

We performed a literature review, the strategy for which is presented in Figure 2. The clinical characteristics of 24 patients, including our patient, are shown in Table 2 [8-29]. The median age of the patients was 68 years with 62.5% men. In 78.2% of patients, the colon was the primary infected GI site. The median period since dialysis began (dialysis vintage) was 48 months. The main symptoms of CMV gastroenterocolitis were abdominal/epigastric pain (54.1%), hematochezia (50.0%), and diarrhea (33.3%). The median time delay from symptom onset to definitive biopsy was 29 days. Of the 24 patients, one was excluded because of missing data. Twenty patients were treated with ganciclovir (GCV), two received VGCV, and one required a switch from GCV to VGCV. In one of the 20 GCV-treated patients, administration was interrupted because of pancytopenia. Regarding the prognosis, as mentioned during the follow-up period, the mortality rate was 18.1%.

**Table 2:** A literature review on CMV enteritis in patients on hemodialysis and the case in this study [8-29] CKD: chronic kidney disease; HD: hemodialysis; PD: peritoneal dialysis; DM: diabetes mellitus; ADPKD: autosomal dominant polycystic kidney disease; HTN: hypertension; HBV: hepatitis B virus; COPD: chronic obstructive pulmonary disease; AA: Amyloid A; CABG: coronary artery bypass graft; PAF: paroxysmal atrial fibrillation; WBC: white blood cell; Ly: lymphocyte count; CRP: C-reactive protein; Hb: hemoglobin; GCV: ganciclovir; VGCV: valganciclovir. *The chief complaint is referred to as “gastrointestinal bleeding” in the paper and is interpreted as hematochezia since the colon is mentioned as the infected site.

Case No	Author	Age (years)	Sex	Type of dialysis	Dialysis vintage (months)	Underlying diseases	Chief complains	Time lag from developing symptoms to the diagnosis (days)	Diagnostic method	Infected sites	WBC (×10^9^/L)	CRP (mg/dL)	Hb (g/dL)	Complications	Antiviral therapy (+/-)	Therapeutic duration (weeks)	Outcome	Recurrence (+/-)
1	Esforzado et al. (1993) [8]	74	Male	HD	1	DM, gastric ulcer	Hematochezia*		Biopsy	Colon	20.5 (Ly 11%)				(+: GCV)	3	Survived	
2	Esforzado et al.(1993) [8]	59	Female	HD	24	DM, right food distal amputation due to peripheral vascular disease	Hematochezia, fever, abdominal pain, diarrhea		Biopsy	Colon	18.1				(+)		Survived	(-)
3	Falagas et al. (1996) [9]	57	Male	HD	6	ADPKD, HTN, nephrolithiasis, asthma, gout, HBV career	Hematochezia, abdominal pain, diarrhea		Biopsy	Colon	10.8 (Ly 7%)		11.5	Hemicolectomy	(+: GCV)	2	Survived	(-)
4	Liu et al. (2018) [10]	77	Female	HD		Type 2 DM, HTN, atrial fibrillation, congestive heart failure	Loss of appetite, nausea, epigastric pain		Biopsy	Stomach	5.3	1.9	6.2		(+: VGCV)	2	Survived	
5	Kim et al. (2011) [11]	56	Male	HD	24	Type 2 DM	Watery diarrhea, abdominal pain	14	Biopsy	Colon	13.2	30	9.1		(+: GCV)	4	Survived	
6	Rankin et al. (2009) [12]	72	Male	HD		COPD, type 2 DM, cardiovascular disease	Hematochezia		Biopsy	Colon	20.2	42	9.5		(+: GCV)	3	Survived	
7	Sudcharoen et al. (2016) [13]	72	Male	HD		Ischemic heart disease, cerebrovascular disease, HTN, gout	Hematochezia	56	Biopsy	Colon					(+: GCV)	1		
8	Tabernero et al. (2004) [14]	72	Male	HD	36	Renal tuberculosis	Fever, hematochezia, abdominal pain	21	Biopsy	Colon	2.7		9.5		(+: GCV)	3	Survived	
9	Hsieh et al. (2015) [15]	59	Female	PD	60		Fever, abdominal pain	85	Biopsy	Colon	17.5				(+: GCV)	3	Survived	
10	Huang et al. (2017) [16]	44	Male	HD		DM	Hematochezia, shock, abdominal pain, fever	13	Biopsy	Colon				Right-colon ischemia with pneumatosis intestinalis, hemicolectomy	(+: GCV)	12	Survived	
11	Quintana et al. (2005) [17]	65	Male	PD	72	Type 2 DM, chronic neurovascular complications	Vomiting, dysphagia	29	Biopsy	Esophagitis					(+: GCV)	2	Survived	
12	Lo (2017) [18]	70	Female	HD	70	Coronary artery disease	Hematemesis		Gastric fluid	Stomach				Hyperkalemia, atrial flutter	(+: GCV → VGCV)			
13	Yap et al. (2016) [19]	52	Male	HD		HTN	Hematochezia, abdominal pain		Biopsy	Small intestine			5.0		(+: GCV)			
14	Li et al. (2015) [20]	83	Female	HD		DM	Hematochezia								(+: GCV)		Survived	
15	Isekawa et al. (2013) [21]	76	Female	HD	144	DM	Hematochezia, diarrhea		Biopsy	Colon					(+: GCV)	4	Survived	
16	Imoto et al. (2010) [22]	65	Male	HD	72	Chronic nephritis, AA amyloidosis	Abdominal pain, diarrhea		Biopsy	Colon					(+: GCV)		Died	
17	Okada et al. (2012) [23]	72	Female	HD	36	DM	Abdominal pain		Biopsy	Colon	16.1	9.7	15.6	Necrosis of the intestine, colectomy, colostomy	(-)		Survived	
18	Asai et al.(2009) [24]	76	Male	HD	144	Chronic glomerulonephritis, CABG, PAF, cervical spondylotic myelopathy	Fever, abdominal pain, diarrhea		Biopsy	Colon					(+: GCV)		Survived	
19	Bando et al. (2008) [25]	72	Male	HD	216	Renal sclerosis	Fever, abdominal pain	38	Biopsy	Colon		22		Fungal abscess of the iliopsoas muscle	(+: GCV)		Survived	
20	Miyauchi et al. (2006) [26]	74	Male	HD	1	Gastric cancer, prostatic cancer, empyema, lung aspergillosis	Vomiting, hematemesis	28	Biopsy	Esophagus	15.8	7.7	3.85		(+: GCV, stopped due to pancytopenia)		Died	
21	Sasaki et al. (2004) [27]	68	Female	HD	60	Tuberculosis pleurisy	Abdominal pain	84	Biopsy	Duodenum	7.1	7.9	8.7		(+: GCV)		Survived	
22	Nishimoto et al. (2002) [28]	57	Female	PD	48	Cholecystectomy, acute pyelonephritis	Watery diarrhea, vomiting		Biopsy	Colon, esophagus, stomach		13.4	5.4	AA amyloidosis	(+: GCV)	5.6	Died	
23	Dayasu et al. (1999) [29]	63	Male	HD	12		Hematochezia	37	Biopsy	Colon					(+: GCV)		Died	
24	The present case	46	Male	HD	108	DM, shunted stenosis	Diarrhea, hematochezia	11	Biopsy	Colon	14.6	2.4	12.7		(+: VGCV)	8	Survived	(-)

Our study supports the hypothesis that dialysis in patients with ESRD is a risk factor for CMV infection. One case-control study did not identify renal disease requiring HD as an independent risk factor for CMV colitis in immunocompetent patients (odds ratio: 1.72, 95% CI: 0.24-12.22, P-value: 0.58) but suggested that red blood cell transfusion within 1 month of diagnosis of colitis was a risk factor [1]. This could explain why dialysis patients receiving frequent transfusions have a higher CMV infection risk. As mentioned previously, another retrospective cohort study identified CKD as a risk factor for CMV enteritis [4]. More specifically, CKD and ESRD led to dendritic cell depletion and dysfunction, reduced CD4/CD8 ratio, increased Th1/Th2 ratio, and depleted naïve and central memory CD4+ and CD8+ T cells [30]. Therefore, it remains controversial whether CMV infection of the GI tract is correlated with dialysis. Our literature review was unable to reveal the detailed history of previous blood transfusion or CD4/CD8 ratio among dialysis patients and prompts further prospective studies.

Furthermore, one retrospective cohort study on GI tract CMV disease showed that the most frequent sites of infection among 89 immunocompetent patients were the colon and rectum (66.3%), followed by the esophagus (5.6%), stomach (4.5%), and small intestine (19.1%) [6]. These results are similar to those of our review. Another literature review of CMV colitis in immunocompetent patients without chronic renal failure [31], reported that the most common presenting symptoms were fever (76%), abdominal or rectal pain (53%), hematochezia (53%), and watery diarrhea (29%). In our study, the symptoms observed in dialysis patients with CMV infection were consistent with these two studies. Additionally, our review revealed that CMV gastroenterocolitis often affects the colon and is accompanied by hematochezia or watery diarrhea. Since CMV diagnosis should be confirmed by a colon biopsy, our findings suggest that if dialysis patients present with hematochezia or watery diarrhea, a colonoscopy should be performed.

Regarding treatment, although low creatinine clearance (<20 mL/min) was reported to be a risk factor for GCV-induced thrombocytopenia [32], the cessation of GCV or VGCV treatment due to thrombocytopenia was not observed in our review, except for one patient with pancytopenia. Therefore, HD-dependent CKD may not increase the incidence of thrombocytopenia and further studies are required to evaluate adverse events associated with GCV or VGCV use in HD patients.

In our review, the median time delay from symptom onset to a definitive biopsy was 29 days, which could be attributed to several reasons. First, CMV enterocolitis is often self-limiting [33,34], and patients can be cured before being diagnosed. Second, it is uncommon for clinicians to suspect CMV infection in immunocompetent patients. Finally, clinicians may not perform a biopsy of a suspicious lesion in some cases, preventing CMV detection at an earlier stage.

A limitation of our study is that the literature review methodology can have reporting bias, lack of generalizability, and a large amount of missing data. Thus, prospective studies are required to confirm the relationship between ESRD with HD and CMV infections of the GI tract in the future.

## Conclusions

To our knowledge, this is the first literature review on CMV infections of the GI tract in patients on HD. Hematochezia and diarrhea were observed as the likely symptoms that increase the pretest probability of CMV reactivation in the GI tract among HD patients. Our analysis suggests that clinicians should suspect CMV infection of the GI tract in dialysis patients, who experience unexplained bloody diarrhea, and promptly perform GI endoscopy and biopsy.
